# Challenges to equitable access to urogenital care in urban and rural Kazakhstan: a mixed-methods study

**DOI:** 10.3389/fpubh.2026.1813983

**Published:** 2026-04-22

**Authors:** Nurgul Darmen, Gulshara Aimbetova, Maiya Zhakupova, Akmaral Aitmanbetova, Adilya Shakhiyeva, Yernur Zikiriya, Nurgul Alekenova

**Affiliations:** 1 Marat Ospanov West Kazakhstan Medical University, Aktobe, Kazakhstan; 2 Asfendiyarov Kazakh National Medical University, Almaty, Kazakhstan; 3 Kazakhstan Medical University “KSPH”, Almaty, Kazakhstan

**Keywords:** genitourinary diseases, health equity, patient satisfaction, primary healthcare, urban and rural areas

## Abstract

**Background:**

Addressing urban–rural inequalities is essential for universal health coverage. This study examined disparities in access to genitourinary (GU) care in Kazakhstan.

**Methods:**

A cross-sectional mixed-methods study was conducted in urban and rural areas of Almaty region (2024–2025). Quantitative data (*n* = 721) were analyzed using chi-square tests and logistic regression. Qualitative interviews with urologists (*n* = 11) were thematically analyzed.

**Results:**

Sex distribution was similar. Urban residents had higher education, while rural residents reported better financial status. Most sought initial care at PHC, though self-treatment was common. Rural residents relied more on PHC and had longer waiting times; urban residents more often used private services. Workforce shortages and limited diagnostic capacity were key barriers, especially in rural areas.

**Conclusion:**

Urban–rural disparities in GU care hinder progress toward universal health coverage in Kazakhstan. Strengthening rural workforce and diagnostics is needed.

## Introduction

Genitourinary (GU) diseases encompass a wide range of conditions affecting the urinary and genital organs and represent a growing public health concern influenced by age, sex, non-communicable diseases, and emerging factors such as intestinal microbiota dysbiosis (1, 2).

Between 1990 and 2019, the global incidence increased for kidney (29.1%), bladder (4%), prostate (22%), and testicular (45.5%) cancers, correlating positively with the socio-demographic index, high body mass index became the leading risk factor for kidney cancer, while smoking remained predominant for bladder and prostate cancers (3). Urinary tract infections (UTIs) affect over 150 million people annually, with healthcare-associated UTIs—largely catheter-related—occurring in 1.4–5.1% of patients and rising to 5–14% in urology units, most commonly presenting as cystitis, pyelonephritis, or sepsis (4, 5).

Achieving universal health coverage (UHC), a core goal of Sustainable Development Goal 3, requires equitable access to health services for both urban and rural populations. Evidence shows persistent urban–rural disparities in urological care, affecting screening, treatment, and survival, largely driven by travel time, urologist density, staffing levels, and the availability of specialized resources (6–8). These disparities can be understood within established theoretical frameworks, such as Andersen’s behavioral model of healthcare utilization and the social determinants of health approach, which emphasize the role of socioeconomic status, geographic accessibility, and health system capacity in shaping access to care. Recent empirical studies further highlight that environmental and structural determinants significantly influence health outcomes and access to medical services in developing regions, underscoring the importance of systemic factors in health inequalities.

Kazakhstan is dedicated to implementing UHC and enhancing primary health care (PHC). As part of PHC development in the Almaty region, the PHC Demonstration Platform World Health The organization was launched, with the goal of improving interdisciplinary care and fostering the exchange of knowledge and best practices within Kazakhstan (9, 10). Between 2015 and 2022, the prevalence of GU in Kazakhstan ranged from 1619.8 to 4984.8 cases per 100,000 people, peaking in 2018, with a projected decline to 1888.3 cases by 2029 (11). Moreover, treatable mortality from renal failure increased from 6.76 to 11.14 per 100,000 population, underscoring the need for improved prevention and treatment, particularly in regions experiencing rising treatable deaths (12). Similar patterns of urban–rural inequality have been documented in Eastern Europe and Central Asia, where disparities in workforce distribution and infrastructure continue to affect access to specialized services.

Despite these trends, there is a lack of empirically and theoretically grounded research examining disparities in access to GU healthcare services in Kazakhstan, particularly from a mixed-methods perspective. Addressing this gap is important not only for describing inequalities but also for understanding the mechanisms through which they are produced and maintained within the healthcare system.

This study aims to compare access to and satisfaction with GU care in the Almaty region, focusing on healthcare utilization, waiting times, referral pathways, and patient satisfaction. We hypothesize that rural residents experience reduced access to specialized services, longer waiting times, and greater reliance on primary healthcare compared to urban populations, reflecting structural and systemic constraints. The findings are expected to contribute both empirically, by providing new evidence from Kazakhstan, and theoretically, by applying established frameworks of healthcare access to a previously underexplored context, thereby informing targeted policy interventions.

## Materials and methods

### Study design and setting

A mixed-methods study was conducted in Almaty oblast, including both urban and rural areas, during 2024–2025. This design was chosen to provide a comprehensive understanding of access to and satisfaction with genitourinary (GU) care by integrating quantitative and qualitative data.

### Quantitative part

A structured questionnaire was developed in Kazakh and Russian languages, which was reviewed and validated by experts in the field of management, urologists and public health. The questionnaire consisted of four sections, such as Demographic characteristics; Experience and Pathways to GU care; Satisfaction and Preferences for GU care. The instrument was pre-tested with 15 respondents to assess clarity, comprehensibility, and cultural appropriateness. Minor adjustments were made based on feedback.

### Sampling and data collection

The questionnaire was distributed in paper version by researchers in urban and rural PHC facilities of the Almaty region. Before we get permission for data collection from healthcare department of the Almaty region. Data collected during second part of the 2024. The inclusion criteria for the study were individuals aged 18 years and older who had been diagnosed with a GD condition and had visited a urologist at a PHC at least once within the past year. The general population was defined based on regional statistics, which reported an average of 79,676 individuals who sought medical care for GD during 2022–2023. Using this population size, the minimum required sample size was calculated to be 384 respondents to ensure adequate statistical power. To account for potential data loss or errors, the study initially aimed to include 768 participants. After data cleaning and validation, 47 questionnaires were excluded due to incomplete or inconsistent responses. As a result, 721 questionnaires were included in the final analysis. Before beginning the survey, respondents were informed about the purpose of the study, assured of the anonymity of their responses, and asked to provide their consent to participate. Additionally, the survey included the researchers’ contact information, allowing participants to reach out with questions, request the results, or discuss any related concerns.

### Statistical analysis

The analysis provide comparison between urban and rural area using the chi-squared test (*χ*^2^) was applied for categorical data expressed as numbers and percentages to assess associations between variables.

Multiple logistic regression used to analyse the satisfaction of the respondents with the GD care where the independent variables was education; gender; place where they received care; type of organization; rate of quality of care. Model diagnostics included: Goodness-of-fit assessed using the Hosmer–Lemeshow test; Multicollinearity checked using variance inflation factors (VIF). A *p*-value <0.05 was considered statistically significant.

Survey data were analyzed using the Statistical Package for the Social Sciences (SPSS, version 23, IBM Corporation, Armonk, NY, USA).

### Qualitative component

#### Data collection

Semi-structured interviews were conducted with urologists working in primary and hospital settings in both urban and rural areas. Sampling continued until thematic saturation was achieved. Interview topics included: Accessibility of urological services; Referral pathways; Role of general practitioners; Availability of diagnostics; Preventive services; Workforce challenges.

Interviews were conducted in Kazakh or Russian, recorded with consent, and transcribed verbatim.

#### Data analysis

A thematic analysis approach was applied following standard qualitative research procedures. Transcripts were independently coded by at least two researchers; an initial coding framework was developed inductively from the data; Codes were grouped into categories and overarching themes through iterative comparison; Discrepancies were resolved through discussion and consensus.

To enhance rigor: Inter-coder reliability was ensured through independent coding and agreement checks; Reflexivity was maintained during analysis; Data saturation was documented.

#### Integration of quantitative and qualitative data

Findings from both components were integrated at the interpretation stage using a triangulation approach. Quantitative results identified patterns and associations, while qualitative findings provided contextual explanations and deeper insights into observed trends, particularly regarding barriers to access and patient satisfaction.

#### Ethical approval

The study was approved by the Local Ethics Committee of the West Kazakhstan Medical University (No. 186, 12-12-2022) and in 2-2025/048-NS, Aktobe, Kazakhstan.

## Results

Sex distribution was similar in urban and rural areas. Urban residents included a higher proportion of pensioners, whereas rural areas had more non-working individuals. Higher education was more common in urban areas (55.6% vs. 44.4%, *p* = 0.016). Rural residents more frequently rated their financial status as good (56.3% vs. 43.7%), while urban residents more often reported it as satisfactory (55.3% vs. 44.7%, *p* = 0.012) (Table 1).

**Table 1:** Demographic characteristics of survey participants.

Variable		Urban (*n*-%)	Rural (*n*-%)	Total (*n*-%)	*p*
Gender	Male	212 (50.6)	207 (49.4)	419 (58.1)	0.806
	Female	150 (49.7)	152 (50.3)	302 (41.9)	
	Total	362 (50.2)	359 (49.8)	721 (100.0)	
Age	18–45	161 (49.5)	164 (50.5)	325 (45.1)	0.054
	46–63	105 (45.7)	125 (54.3)	230 (31.9)	
	Over 63	96 (57.8)	70 (42.2)	166 (23.0)	
Working status	Working	237 (50.6)	231 (49.4)	468 (64.9)	<0.001
	Not working	43 (36.1)	76 (63.9)	119 (16.5)	
	Pensioners	82 (61.2)	52 (38.8)	134 (18.6)	
Education	Higher	164 (55.6)	131 (44.4)	295 (40.9)	0.016
	Secondary	198 (46.5)	228 (53.5)	426 (59.1)	
Self-assessment of financial situation	Good	129 (43.7)	166 (56.3)	295 (40.9)	0.012
	Satisfactory	208 (55.3)	168 (44.7)	376 (52.1)	
	Unsatisfactory	25 (50.0)	25 (50.0)	50 (6.9)	

Urban residents were more likely to initially seek care at private clinics (65.6%), whereas rural residents were more often contacted local PHC facilities (*p* = 0.05). About one-third of participants in both areas reported self-treatment (30.5%). Urban residents more frequently used private medical organizations overall (81.8% vs. 18.2%), while rural residents were more likely to visit a local urologist at PHC (64.6%, *p* < 0.001). Rural participants reported more frequent visits (>4 times), whereas urban residents typically visited once or twice (*p* < 0.001). Diagnostic tests were more commonly performed in private facilities among urban residents (67.8% vs. 32.2%, *p* < 0.001). Most received appointments in both regions within 1–7 days (88.7%), although some rural residents waited 7–14 days. Rural residents were more often referred to urologists by other physicians (15.0% vs. 6.4%), while urban residents more frequently self-referred (16.0% vs. 12.5%, *p* = 0.003). A higher proportion of urban residents received free medications at PHC facilities (59.8% vs. 40.2%, *p* = 0.05) (Table 2).

**Table 2:** Experience and pathways to urological care: a comparison of urban and rural populations.

Variable		Urban (*n*-%)	Rural (*n*-%)	Total (*n*-%)	*p*
When the first signs GD appear, you	Treated themselves	112 (50.9)	108 (49.1)	220 (30.5)	0.051^*^
	Contacted the PHC	180 (47.0)	203 (53.0)	383 (53.1)	
	Called an ambulance	28 (51.9)	26 (48.1)	54 (7.5)	
	Contacted a private clinic	42 (65.6)	22 (34.4)	64 (8.9)	
For the first time, you sought medical help for a GD	To the GP at the PHC	185 (48.4)	197 (51.6)	382 (53.0)	<0.001^*^
	To the urologist at the PHC	64 (35.4)	117 (64.6)	181 (25.1)	
	To the nurse at the PHC	27 (67.5)	13 (32.5)	40 (5.5)	
	To the ambulance	32 (61.5)	20 (38.5)	52 (7.2)	
	To the doctor at a private clinic	54 (81.8)	12 (18.2)	66 (9.2)	
Under what circumstances were you diagnosed with a GD	Due to illness	280 (50.6)	273 (49.4)	553 (76.7)	0.149
	Medical examination	65 (47.4)	72 (52.6)	137 (19.0)	
	Injury or accident	10 (43.5)	13 (56.5%)	23 (3.2)	
	Need to obtain a certificate	7 (87.5)	1 (12.5)	8 (1.1)	
Indicate the frequency of visits to a doctor regarding a GD during the year	1 time	199 (52.6)	179 (47.4)	378 (52.4)	<0.001^*^
	2–3 times	143 (52.8)	128 (47.2)	271 (37.6)	
	More than 4 times	20 (27.8)	52 (72.2)	72 (10.0)	
You sought medical help	Private clinic	94 (68.6)	43 (31.4)	137 (19.0)	<0.001^*^
	PHC	268 (45.9)	316 (54.1)	584 (81.0)	
Where did you undergo diagnostic and laboratory tests for your illness	PHC	218 (47.8)	238 (52.2)	456 (63.2)	<0.001^*^
	Private clinic	78 (67.8)	37 (32.2)	115 (16.0)	
	Both	66 (44.0)	84 (56.0)	150 (20.8)	
How many days does it take to wait for an appointment with a urologist from the moment of registration	1–3 days	188 (47.4)	209 (52.6)	397 (55.1)	0.024^*^
	4–7 days	139 (57.4)	103 (42.6)	242 (33.6)	
	8–14 days	26 (41.9)	36 (58.1)	62 (8.6)	
	15–30 days	4 (30.8)	9 (69.2)	13 (1.8)	
	More than 30 days	5 (71.4)	2 (28.6)	7 (1.0)	
Which specialists referred you to the urologist	GP	198 (51.0)	190 (49.0)	388 (53.8)	0.003^*^
	Therapist	54 (56.8)	41 (43.2)	95 (13.2)	
	Emergency medical doctor	29 (50.0)	29 (50.0)	58 (8.0)	
	Appealed independently	58 (56.3)	45 (43.7)	103 (14.3)	
	Other doctors	23 (29.9)	54 (70.1)	77 (10.7)	
How much time passed to visit urologist	Less than 7 days	296 (52.6)	267 (47.4)	568 (78.1)	0.022^*^
	7–14 days	42 (38.2)	68 (61.8)	110 (15.3)	
	More than a 15 days	24 (50.0)	24 (50.0)	48 (6.7)	
Do you receive free medications for GD (yes answer) at PHC		52 (59.8)	35 (40.2)	87 (12.1)	0.057

GD, genitourinary system disease. * Indicates significant values of *p*.

Overall satisfaction was high across both regions. Most respondents were fully satisfied with appointment scheduling (68.9%) and availability of diagnostic tests (69.0%) (not significant). About 66.6% were fully satisfied with waiting times, with rural residents reporting slightly higher satisfaction than urban residents (*p* = 0.05). Satisfaction with medical staff care was high (72.9%), particularly among rural participants (54% vs. 46%, *p* < 0.001). Similarly, 77.4% were satisfied with information about their health status, again higher in rural areas (54.3% vs. 45.7%, *p* < 0.001). Rural respondents also reported greater satisfaction with nursing care (*p* = 0.014), waiting time for planned hospitalization (*p* < 0.001), quality of GP care (*p* = 0.006), and urological care at PHC (52.2% vs. 45.8%, *p* < 0.001). In contrast, urban residents were more likely to express a positive opinion about online urology consultations (56.0% vs. 44.0%, *p* = 0.012) (Table 3).

**Table 3:** Satisfaction and preferences for urological care: comparison between urban and rural populations.

Variable		Urban (*n*-%)	Rural (*n*-%)	Total (*n*-%)	*p*
Making an appointment with a doctor	Fully satisfied	247 (49.7)	250 (50.3)	497 (68.9)	0.910
	Partially satisfied	93 (51.1)	89 (48.9)	182 (25.2)	
	Not satisfied	22 (52.4)	20 (47.6)	42 (5.8)	
Waiting times for diagnostic tests after making an appointment	Fully satisfied	227 (47.3)	253 (52.7)	480 (66.6)	0.057^*^
	Partially satisfied	120 (57.1)	90 (42.9)	210 (29.1)	
	Not satisfied	15 (48.4)	16 (51.6)	31 (4.3)	
Availability of diagnostic tests	Fully satisfied	241 (48.7)	254 (51.3)	495 (69.0)	0.101
	Partially satisfied	101 (51.0)	97 (49.0)	198 (27.6)	
	Not satisfied	17 (70.8)	7 (29.2)	24 (3.3)	
Care for patients and interest in their problems on the part of medical staff	Fully satisfied	241 (46.0)	283 (54.0)	524 (72.9)	0.001^*^
	Partially satisfied	109 (63.7)	62 (36.3)	171 (23.8)	
	Not satisfied	12 (50.0)	12 (50.0)	24 (3.3)	
Obtaining information about your health status, diagnosis and treatment	Fully satisfied	255 (45.7)	303 (54.3)	558 (77.4)	0.001^*^
	Partially satisfied	98 (67.1)	48 (32.9)	146 (20.2)	
	Not satisfied	9 (52.9)	8 (47.1)	17 (2.4)	
Quality of medical care provided by nurses	Fully satisfied	284 (47.7)	311 (52.3)	595 (82.5)	0.014^*^
	Partially satisfied	73 (62.4)	44 (37.6)	117 (16.2)	
	Not satisfied	5 (55.6)	4 (44.4)	9 (1.2)	
Organization of the work of the PHC	Fully satisfied	230 (47.9)	250 (52.1)	480 (66.6)	0.185
	Partially satisfied	115 (55.6)	92 (44.4)	207 (28.7)	
	Not satisfied	17 (50.0)	17 (50.0)	34 (4.7)	
Waiting periods for planned hospitalization	Fully satisfied	244 (45.2)	296 (54.8)	540 (74.9)	0.001*
	Partially satisfied	100 (64.9)	54 (35.1)	154 (21.4)	
	Not satisfied	18 (66.7)	9 (33.3)	27 (3.7)	
Results of treatment, examination or rehabilitation of a urologist	Fully satisfied	302 (49.7)	306 (50.3)	608 (84.3)	0.537
	Partially satisfied	57 (52.3)	52 (47.7)	109 (15.1)	
	Not satisfied	3 (75.0)	1 (25.0)	4 (0.6)	
Rate the quality of medical care provided to you by a GP on a 5-point scale	Fully satisfied	163 (45.7)	194 (54.3)	357 (49.5)	0.006^*^
	Partially satisfied	146 (58.6)	103 (41.4)	249 (34.5)	
	Not satisfied	41 (44.1)	52 (55.9)	93 (12.9)	
	Fully satisfied	8 (72.7)	3 (27.3)	11 (1.5)	
	Partially satisfied	4 (36.4)	7 (63.6)	11 (1.5)	
If you visited a urologist at a PHC, rate the quality of medical care on a 5-point scale	Not satisfied	260 (45.8)	308 (54.2)	568 (78.8)	<0.001^*^
	Fully satisfied	77 (69.4)	34 (30.6)	111 (15.4)	
	Partially satisfied	16 (59.3)	11 (40.7)	27 (3.7)	
	Not satisfied	6 (66.7)	3 (33.3)	9 (1.2)	
	Fully satisfied	3 (50.0)	3 (50.0)	6 (0.8)	
How do you feel about online consultation with a urologist (those who responded positively)		159 (56.0)	125 (44.0)	284 (39.4)	0.012*

* Indicates significant values of *p*.

The logistic regression analysis was conducted to examine factors influencing healthcare utilization and satisfaction with GD care. The results showed that individuals who contacted PHC had 45% higher odds of receiving treatment compared to those who self-treated (AOR = 1.45), suggesting that formal entry into the healthcare system improves access to care. Similarly, individuals who called an ambulance had more than twice the odds of receiving medical care (AOR = 2.06), indicating that emergency pathways facilitate timely access to services. Furthermore, individuals who preferred government services were more likely to be satisfied with government care compared to private care (AOR = 1.68), which may reflect greater affordability and continuity of care. Individuals not registered with a dispensary group at PHC had slightly higher odds of seeking healthcare (AOR = 1.52), possibly indicating gaps in regular follow-up. Additionally, individuals who rated their care as “good” had significantly lower odds of seeking further treatment compared to those rating it as “excellent” (AOR = 0.24), suggesting that the higher perceived quality of care may reduce the need for additional healthcare use (Table 4).

**Table 4:** Logistic regression analysis to examine factors influencing satisfaction with healthcare for diseases of the genitourinary system.

Variable		Odds ratio	Adjusted odds ratio
Place	Rural	1	1
	Urban	1.06 (075; 1.49)	1.21 (0.84; 1.75)
Education	Higher	1	1
	Secondary	1.08 (0.77; 1.53)	1.10 (0.77; 1.59)
Age	18–45	1	1
	46–63	1.20 (0.81; 1.78)	1.07 (0.70; 1.63)
	over 63	1.21 (0.78; 1.88)	1.17 (0.72; 1.88)
Gender	Male	0.84 (0.59; 1.18)	1.16 (0.80; 1.66)
	Female	1	1
Get treatment	Treated themselves	1	1
	Contacted the PHC	1.46 (1; 2.15)	1.45 (0.97; 2.18)
	Called an ambulance	1.96 (0.91; 4.25)	2.06 (0.92; 4.59)
	Contacted a private clinic	0.61 (0.34; 1.1)	0.61 (0.33; 1.13)
Which medical organization would you prefer to contact?	Private	1	1
	Government	1.99 (1.36; 2.91)	1.68 (1.11; 2.53)
Are you registered with a dispensary group in PHC	Yes	1	1
	No	1.37 (0.93; 2)	1.52 (1.02; 2.27)
Rate the quality of medical care provided to you on a 5-point scale	Excellent	1	1
	Good	0.31 (0.05; 1.85)	0.24 (0.84; 1.51)
	Rather good	0.62 (0.15; 2.5)	0.65 (0.15; 2.78)
	Bad	1.04 (0.27; 4.04)	1.03 (0.25; 4.28)
	Very bad	1.62 (0.42; 6.28)	1.44 (0.35; 5.93)

### Qualitative findings

Analysis of responses from eleven participants indicated that urological care is generally perceived as satisfactory. However, it has consistently identified systemic challenges, particularly in rural areas and districts with large populations served. Although basic urological services are formally available, structural and organizational barriers limit their effectiveness.

### Accessibility of care

Access to urologists is formally ensured in most outpatient clinics, with waiting times ranging from 2–3 days to 1–2 weeks. However, accessibility is constrained by high specialist workload, part-time employment, and high patient volumes. Ineffective referral pathways further delay care, as patients are frequently referred without complete diagnostic investigations. Respondents also noted that emergency access is particularly limited in remote areas, were delayed presentation results in postponed treatment.

### Role of general practitioners (GP)

Participants reported limited involvement of GPs in the diagnosis and management of urological conditions. Although GPs are authorized to treat uncomplicated cases, insufficient training and excessive workload lead to over-referral to urologists, reducing the effectiveness of urological care at the PHC level.

### Equipment and diagnostics

Inadequate equipment and diagnostic capacity were identified as major barriers. Respondents highlighted the absence of cystoscopes, urodynamic equipment, and facilities for minor procedures, as well as limited access to ultrasound, computer tomography, and Magnetic Resonance Imaging. These limitations contribute to prolonged waiting times and increased out-of-pocket expenditures, exacerbating inequities in access to care.

### Prevention and follow-up

Preventive activities and dispensary follow-up were largely absent. Patients commonly present with advanced disease, prostate cancer screening is irregular, and structured long-term follow-up is not implemented. Preventive efforts are mostly limited to individual counseling during consultations.

### Workforce constraints

All emphasized is a severe shortage of urologists, especially in rural regions. Low salaries, high workload, limited professional development opportunities, and insufficient institutional support were identified as key contributing factors. Participants suggested strengthening GP training, improving infrastructure, optimizing referral pathways, expanding preventive programs, and introducing financial incentives to improve workforce retention.

These findings support the quantitative results, particularly regarding limited access and reliance on PHC in rural areas. Workforce shortages and diagnostic constraints identified in interviews help explain longer waiting times and repeated healthcare visits observed in the survey data.

## Discussion

This mixed-methods study demonstrates persistent urban–rural disparities in access to and utilization of GU care in the Almaty region, highlighting structural barriers to achieving UHC. Although PHC serves as the main entry point for GU services, rural residents experience longer waiting times, delayed referrals, and limited access to specialized diagnostic services, whereas urban residents more frequently rely on private healthcare providers. Access challenges in rural areas are further compounded by long travel distances between districts and seasonal weather conditions, which can delay care and necessitate the maintenance of economically ineffective facilities to ensure timely emergency services. These findings are consistent with international evidence emphasizing the importance of geographic distance, density, and health system capacity in shaping access to urological specialist care in rural settings (8). These findings can be interpreted within established frameworks of healthcare access, such as Andersen’s behavioral model, where access is shaped by predisposing, enabling, and need factors, including socioeconomic status, geographic accessibility, and health system capacity.

The greater use of private clinics among urban residents may reflect better availability, perceived quality, and trust in private providers; however, it also raises concerns regarding financial protection and health system sustainability (13). In Kazakhstan, out-of-pocket health expenditures remain a pressing issue and, despite recent reductions, continue to exceed the World Health Organization’s recommended threshold of 20%, potentially undermining system resilience (14). Although recent reforms transitioning from a Beveridge-type model to a social health insurance system represent an important step toward reducing financial burden, disparities in access and affordability persist. These findings are supported by the qualitative data, which highlight workforce shortages, limited diagnostic capacity, and ineffective referral systems as key barriers.

In contrast, rural residents’ reliance on PHC—often without timely referral to specialists—reveals gaps in diagnostic capacity and workforce preparedness. Limited involvement of GPS in GU disease management, as reported by healthcare providers, further exacerbates these challenges and underscores the need for targeted training, clearer clinical pathways, and stronger integration between PHC and specialized care. Despite these access constraints, rural residents reported higher satisfaction with interpersonal aspects of care, which may be attributed to closer patient–provider relationships and additional government financial allowances for rural populations (15). Previous studies suggest that trust in healthcare providers is influenced by sociocultural factors, including regional context, education, and family ties to the healthcare profession (16, 17). Nevertheless, qualitative findings indicate that this perceived satisfaction coexists with significant systemic limitations, including inadequate equipment, weak preventive and follow-up services, and a critical shortage of urologists, particularly outside urban centers, consistent with findings from other settings (18, 19). These disparities are driven by the interaction of geographic barriers, limited infrastructure, workforce shortages, and financial constraints, which together restrict timely access to specialized care in rural areas. Similar urban–rural disparities have been reported in Eastern Europe and Central Asia, where limited specialist availability and infrastructure gaps continue to affect access to care in peripheral regions.

Kazakhstan has introduced telemedicine primarily to support provider-to-provider consultations across levels of care, such as between district hospitals and regional or national centers. However, structured teleconsultations involving PHC physicians and patients, as well as integrated teleurology services at the PHC level remain underdeveloped. Although rural residents expressed lower interest in online consultations, evidence from other contexts indicates that teleurology and outreach models can improve access, continuity of care, and specialist support when integrated into routine PHC services (19–21).

Strengthening the health workforce should include targeted incentives for rural retention, such as financial support, career development opportunities, and housing programs. Expanding diagnostic infrastructure at the PHC level, implementing standardized referral pathways, and integrating teleurology services into routine care could improve access and reduce delays. In addition, strengthening preventive programs and GP training may enhance early detection and continuity of care.

### Limitation and future steps

This study has several limitations that should be considered when interpreting the results. First, the cross-sectional design captures responses at a single point in time, limiting the ability to infer causal relationships between variables. Second, although efforts were made to ensure representativeness by including both urban and rural participants from a variety of primary care settings, the findings should be interpreted with caution when generalizing to other regions or healthcare systems. The qualitative component, while providing in-depth insights, involved a limited number of providers and may not capture the full range of perspectives. Future research should employ longitudinally and interventional designs to evaluate the impact of teleurology, outreach clinics, and workforce redistribution strategies. Patient-centered qualitative studies could further elucidate barriers to care navigation and unmet needs. Additionally, the high prevalence of self-medication observed in this study highlights the need for stronger regulation of antibiotic sales and public education on responsible medication use to prevent complications and antimicrobial resistance (16). Strengthening policy measures in these areas will be essential to improving the quality, equity, and sustainability of GU care.

## Conclusion

Urban–rural disparities in access to GU care in the Almaty region remain a significant barrier to achieving universal health coverage (UHC). Rural residents face longer waiting times and limited access to specialized services, while urban residents more frequently use private care. Despite similar overall satisfaction, rural patients reported higher satisfaction with interpersonal care, even in the presence of structural constraints. Quantitative and qualitative findings highlight systemic gaps, including limited diagnostic capacity, weak preventive services, insufficient involvement of general practitioners, and a shortage of urologists in rural areas.

From a theoretical perspective, these findings support established frameworks of healthcare access, demonstrating how geographic, organizational, and socioeconomic factors interact to shape service utilization and equity. Empirically, this study provides novel evidence from Kazakhstan, contributing to the limited literature on urogenital care access in Central Asia.

This study has several limitations. The cross-sectional design limits causal interpretation, and the findings may not be fully generalizable beyond the study region. In addition, the qualitative component reflects a limited number of provider perspectives.

Future research should explore longitudinal and comparative approaches to better understand changes in access over time and evaluate the effectiveness of interventions such as telemedicine, outreach services, and workforce redistribution. Further interdisciplinary research is also needed to examine patient behavior and healthcare system performance.

From a policy perspective, reducing regional inequities requires targeted interventions, including strengthening the rural health workforce through financial and professional incentives, expanding diagnostic infrastructure at the PHC level, integrating teleurology into routine care, and improving referral systems. These measures are essential to ensure equitable and sustainable access to urogenital care.

## Data Availability

The raw data supporting the conclusions of this article will be made available by the authors, without undue reservation.
